# Thyroid storm after coronary artery bypass surgery: a case report

**DOI:** 10.1186/s13019-020-1044-2

**Published:** 2020-01-16

**Authors:** Jae Hoon Lee

**Affiliations:** Department of Thoracic and Cardiovascular Surgery, Sanggye Paik Hospital, Inje University School of Medicine, 1342 Dongil-ro, Nowon-gu, Seoul, South Korea

**Keywords:** Thyroid storm, Heart failure, Tachycardia, Coronary artery bypass

## Abstract

**Background:**

Thyroid storm is a rare, life-threatening disease triggered by an acute event or trauma, such as surgery of the thyroid or another area, and infection. However, recent studies have shown that irregular use or discontinuation of antithyroid drugs is the most common cause of thyroid storm. A cardiovascular event caused by thyroid storm following coronary artery bypass graft (CABG) is high output heart failure with extreme tachycardia, which can be fatal. Thyroid storm after nonthyroidal surgery, especially CABG, has been rarely reported, with only one reported case until now. Herein, we present a case of thyroid storm onset in a patient who underwent CABG.

**Case presentation:**

A 74-year-old woman with a history of antithyroid medication discontinuation against medical advice underwent urgent CABG. The patient exhibited extreme tachycardia postoperatively, which is highly suggestive of thyroid storm. Although a higher infection risk is an important consideration, a high-dose steroid was used to control the intractable tachycardia that did not respond to beta-blocker administration. Despite appropriate antibiotic treatment, the patient’s condition was exacerbated, and she developed multiple organ failure resulting from adult respiratory distress syndrome progression, and she died on day 8 after surgery.

**Conclusions:**

Risk factors for thyroid storm after CABG and its treatment outcomes are rarely reported. Patients with a history of inappropriate antithyroid medication prescription should be in a euthyroid state before surgery. If surgery is imminent, anticipating thyroid storm and its treatment as well as a euthyroid state can improve recovery outcomes postoperatively.

## Background

Thyroid storm is a rare, life-threatening disease characterized by severe clinical manifestations of thyrotoxicosis [1]. The incidence of thyroid storm was 0.20–0.76/100,000 persons per year, with 4.8–5.6/100,000 hospitalized patients per year [2, 3]. In a United States survey, 16% of inpatients with thyrotoxicosis were diagnosed with thyroid storm, which was associated with a significantly higher mortality rate than that in patients with thyrotoxicosis without thyroid storm [2]. Thyroid storm may be precipitated due to the irregular use or discontinuation of antithyroid medication and an acute event such as thyroid or nonthyroidal surgery or infection, especially of the upper respiratory tract [2, 3]. One of the cardiovascular events caused by thyroid storm following coronary artery bypass graft (CABG) is high output heart failure with extreme tachycardia, which can be fatal. Thyroid storm after nonthyroidal surgery, especially CABG, has been rarely reported, with only one reported case until now [4]. Herein, we present a case of thyroid storm onset in a patient who underwent CABG.

## Case presentation

A 74-year-old woman was admitted to our hospital because of dyspnea and chest pain for 1 month. She was previously diagnosed with hyperthyroidism due to heart failure with atrial fibrillation 11 years prior, which was treated with medication prescribed at a private clinic. However, she had discontinued taking medication for hyperthyroidism against medical advice several years prior. Two months before the most recent hospital admission, a thyroid function test (TFT) was administered to determine the cause of weight loss, and she was diagnosed with recurring hyperthyroidism; however, she refused treatment with medication.

**Fig. 1:**
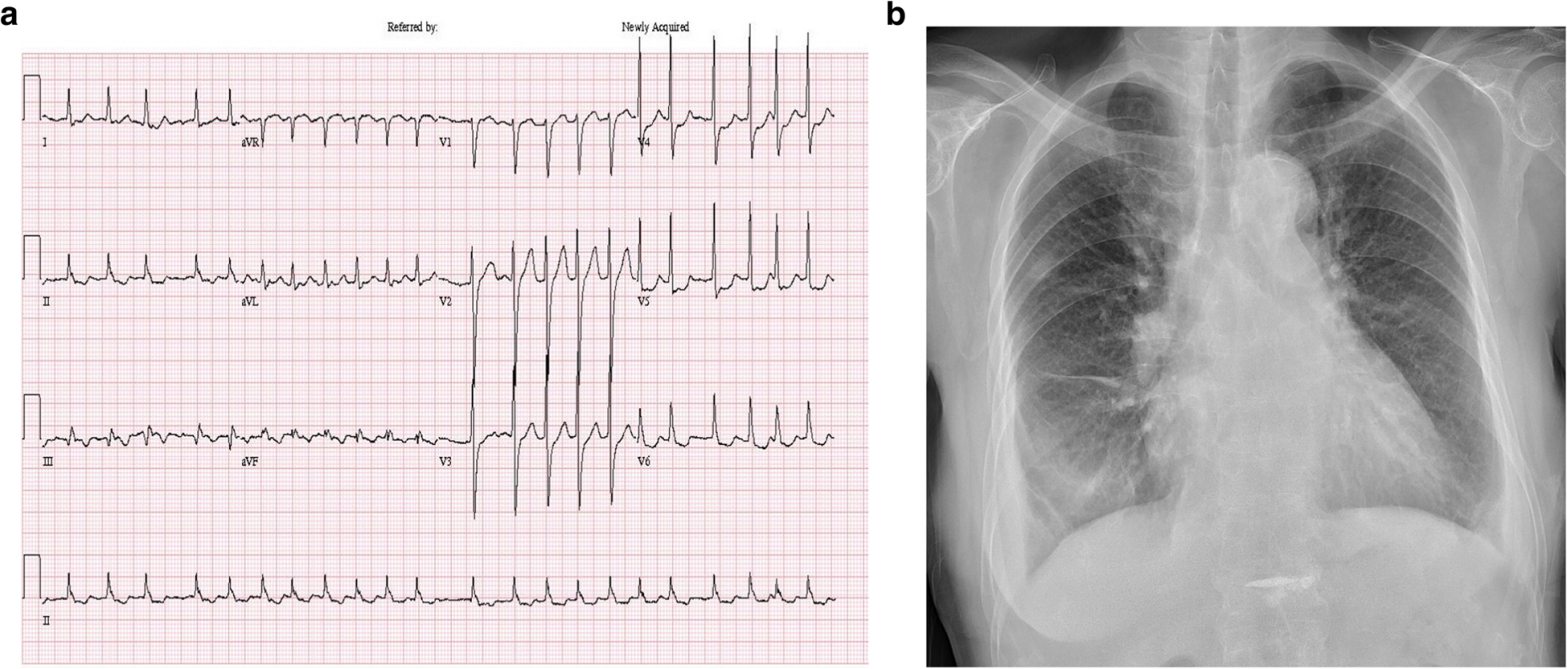
**a** Electrocardiography obtained on admission showing atrial fibrillation. **b** Initial chest roentgenogram showing bilateral pulmonary edema and pleural effusion

Initial chest roentgenography showed bilateral pulmonary edema and pleural effusion, and electrocardiography showed atrial fibrillation (Fig. 1). Transthoracic echocardiography (TTE) showed moderate left ventricle dysfunction (ejection fraction, 43%) with regional wall motion abnormalities at the area of the right coronary artery (RCA) and left circumflex artery (LCX). Subsequent coronary angiography revealed chronic total occlusion (CTO) of the RCA with collateral blood flow from the LCX, 90% obstruction of the LCX, and 80% obstruction in the mid portion of the left anterior descending artery (LAD). Initial cardiac markers showed normal creatine kinase-muscle/brain (CK-MB, 1.8 ng/mL; reference range, < 3.4 ng/mL) and mildly elevated troponin I (Tn I, 0.28 ng/mL; reference range, < 0.12 ng/mL) levels. TFT showed decreased thyroid stimulating hormone (TSH) (0.01 uIU/mL; reference range, 0.55–4.78 uIU/mL) and elevated T3 (2.39 ng/mL; reference range, 0.6–1.81 ng/mL) and free T4 (3.321 ng/dl; reference range, 0.89–1.76 ng/dl) levels and positive test results for thyroglobulin and thyroid peroxidase antibodies.

**Fig. 2:**
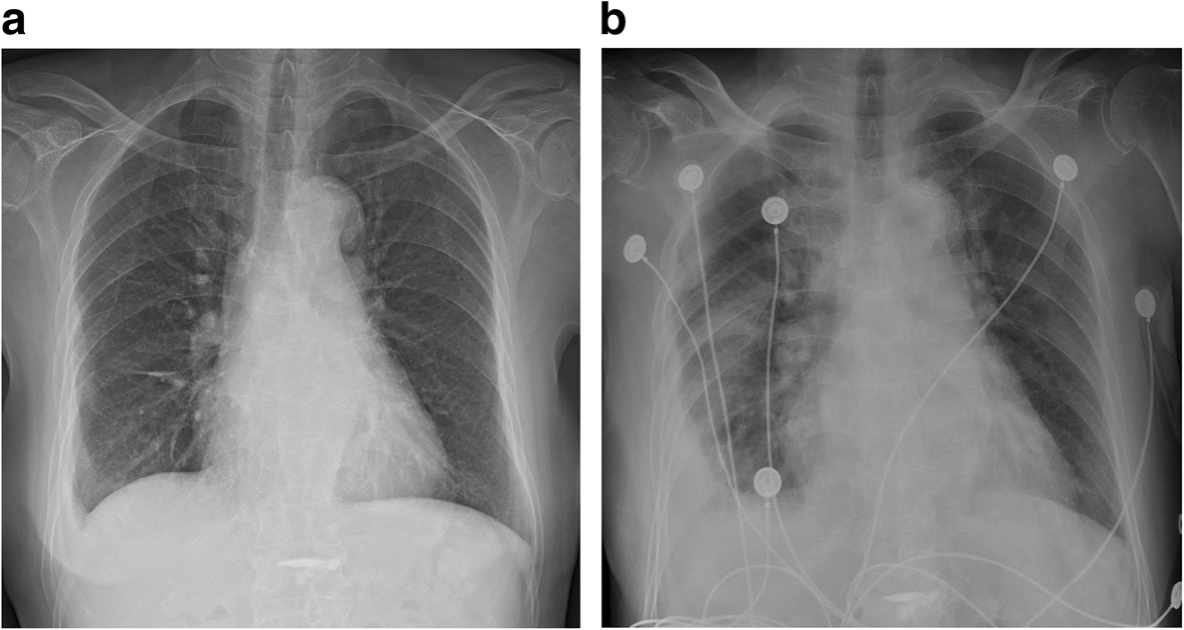
**a** Chest roentgenogram showing improving pulmonary edema and pleural effusion after medical management for 1 week. **b** Follow-up after events of loss of consciousness (LOC) showing aggravation of pulmonary edema with pleural effusion

Anti-thyroid medication treatment with 10 mg methimazole three times per day was initiated, and she was scheduled to undergo CABG surgery after normalization of the thyroid function. Chest roentgenography showed improving pulmonary edema and pleural effusion after medical management for 1 week (Fig. 2a). However, results of the TFT remained elevated (T3, 2.21 ng/ml; free T4, 3.77 ng/dl), and she developed fever (37.9 °C) without any sign of infection.

On day 8 of hospitalization, the patient exhibited loss of consciousness (LOC) with 7 s of sinus pause during the 6-min walk test and then again for 30 s at night. We performed electroencephalography to exclude seizure as a cause, and the findings were normal.

We concluded that sick sinus syndrome (SSS) caused the LOC. A follow-up chest roentgenography showed aggravation of pulmonary edema with pleural effusion and an elevated CK-MB level (8.9 ng/mL; Tn I, 1.82 ng/mL) (Fig. 2b). A subsequent TTE showed no differences in findings compared to those from the initial TTE. We considered delaying CABG surgery because we could not determine whether the patient’s syncope was related to CTO of the RCA.

We decided to perform surgery on the patient after consulting with an endocrinologist who recommended iodine solution administration (Lugol’s solution, 5 drops two times per day) and continuous use of antithyroid medication until surgery. However, results of the TFT remained elevated (T3, 1.89 ng/ml; free T4, 3.64 ng/dl) before surgery. On day 14 of hospitalization, CABG surgery was performed under cardiopulmonary bypass (CPB). The left internal mammary artery was anastomosed to the LAD, and the saphenous vein was anastomosed to the obtuse marginal artery. Graft patency was normal on blood flowmetry. She was successfully weaned from CPB. However, she exhibited extreme tachycardia (> 140 bpm) refractory to the intravenous beta-blocker (esmolol) and was, therefore, transferred to the intensive care unit after the intravenous administration of 130 mg of esmolol bolus injection.

Intravenous esmolol was administered by continuous infusion (range, 20–200 mg/h) and twenty-one 790 mg bolus injections on the day of surgery. In addition, she was administered cortisol (50 mg q 8 h), and the antithyroid medication dosage (15 mg methimazole three times per day) was increased after consultation with the endocrinology department. Twenty-hours after surgery, extreme tachycardia was not present; therefore, continuous infusion and bolus injection of esmolol were discontinued. Follow-up TTE results were the same as those seen preoperatively.

**Fig. 3:**
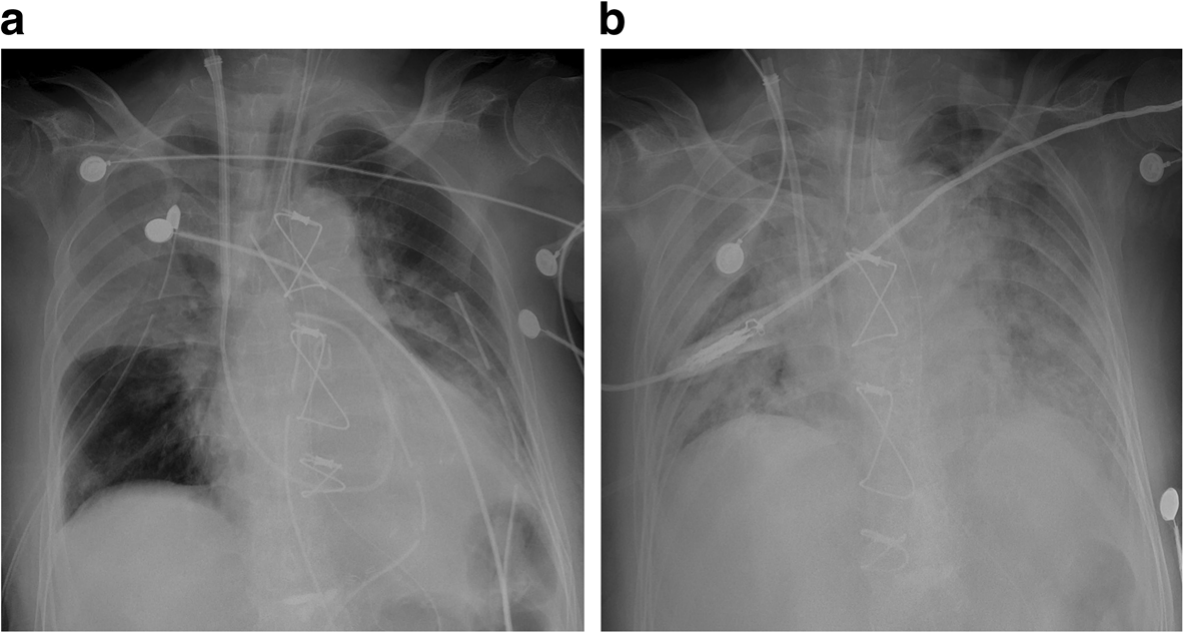
**a** Postoperative chest roentgenogram showing abrupt haziness of the right upper lung field on day 4 after surgery and (**b**) rapid deterioration of adult respiratory distress syndrome (ARDS) on day 8 after surgery

On day 2 after surgery, she exhibited tachycardia (> 160 bpm) for 30 min, which was controlled with five bolus injections of esmolol intravenously (120 mg total). Thereafter, she did not exhibit extreme tachycardia requiring intravenous beta-blocker. The steroid was discontinued on day 3 after surgery for a total of nine bolus injections (450 mg total). Results of the TFT showed decreased thyroid hormone levels (T3, 0.87 ng/ml; free T4, 2.86 ng/dl) on day 4 after surgery. Subsequent chest roentgenography showed haziness of the right upper lung field, and *Klebsiella pneumonia* was detected in the sputum (Fig. 3a). Despite appropriate antibiotic treatment, her condition was exacerbated and she developed multiple organ failure resulting from the deterioration of adult respiratory distress syndrome. She subsequently died 8 days after surgery (Fig. 3b).

## Discussion and conclusions

Thyroid hormone plays an important role in the cardiovascular system and regulates the heart rate, cardiac contractility, myocardial oxygen consumption, and systemic vascular resistance [5].

In patients with hyperthyroidism, various symptoms and signs from cardiovascular origins can present. Palpitation due to sinus tachycardia is typical, and atrial fibrillation appears in 5–15% of patients [5]. Approximately 6% of patients experience heart failure, and most patients with heart failure (94%) have atrial fibrillation. Heart failure symptoms are known to be reduced when thyroid function is normalized, allowing for heart rate control and restored normal sinus rhythm [6].

The pattern of cardiovascular events in patients with thyroid disorder is characterized by high-output heart failure; rarely, if coronary vasospasm causes myocardial ischemia, CABG may be required [7].

Thyroid storm can be triggered by an acute event or trauma, such as surgery involving the thyroid or other areas, infection, and parturition [1–3]. Among the various precipitating factors, a recent study showed that irregular use or discontinuation of antithyroid drugs is the most common factor [3].

If surgery of the thyroid or other area is required, a preoperative euthyroid state can prevent thyroid storm postoperatively. In case of overt hyperthyroidism showing suppressed TSH level with elevated free T4 and/or T3 levels, thionamide administration for 3–8 weeks before performing elective surgery is recommended to ensure that the patient has a normal thyroid function [8]. In urgent cases, the patient should be treated as soon as possible, and, if not contraindicated, beta-blockers for rate control as well as iodine and steroids should also be considered if rapid preparation is required or more severe thyrotoxicosis is present [8]. Treatment is intended to bring the patient as close as possible to a euthyroid state preoperatively.

Our patient showed a history of noncompliance with thyroid medication treatment, overt hyperthyroidism on laboratory results, and heart failure with atrial fibrillation as well as fever without infection. Therefore, thyroid storm onset was very concerning when surgery was performed before the normalization of thyroid hormone.

Initially, we had decided to perform elective surgery after confirmation of a euthyroid state because the patient’s coronary artery disease was caused by atherosclerotic change due to long-standing diabetes mellitus not related to coronary spasm and the patient’s pulmonary edema with pleural effusion improved after treatment of heart failure.

However, we performed surgery before a euthyroid state was confirmed due to aggravation of congestive heart failure and possible deterioration of SSS from coronary artery disease.

The patient’s clinical manifestation of thyroid storm was aggravated after transfer to the intensive care unit. According to the diagnostic criteria of thyroid storm by Burch and Wartofsky, a score of ≥45 is highly suggestive of the condition; we confirmed this diagnosis as our patient’s score was at least 70 [1].

Thyroid storm after nonthyroidal surgery, especially CABG, has been rarely reported. Successful treatment with intravenous beta-blocker and high-dose steroid was reported in a patient without preoperative history of hyperthyroidism [4]. According to the recent suggested guidelines, high-dose steroid treatment was recommended, if necessary, but caution is warranted because of a higher infection risk [9].

We believe that early steroid treatment was inevitable in our patient, considering that the extreme tachycardia, although nonresponsive to beta-blockers, can be fatal in patients who have undergone CABG. However, in elderly patients, the early administration of steroids is associated with multiorgan failure despite appropriate antibiotic therapy.

Although patients requiring CABG usually require immediate surgery depending on their condition, the analysis of risk factors for thyroid storm immediately after surgery and subsequent treatment results have been rarely reported. As this is only a single case, it may not be generalizable and applicable to patients in other circumstances. According to several studies and the outcomes of our patient, it is important that patients with a history of inappropriate antithyroid medication therapy have a euthyroid state before surgery. If surgery is inevitable, preparing for thyroid storm is promising for patient recovery postoperatively.

In conclusion, although our patient died from infection, we believe that this case report is valuable for the planning, prevention, and treatment of thyroid storm in non-thyroidal surgery, especially with emergent heart surgery such as CABG. Further studies are needed to assess the optimal surgical timing and risk factors for nonthyroidal surgery, especially cardiac surgery, in patients with hyperthyroidism.

## Data Availability

Not applicable.

## Abbreviations

CABG: Coronary artery bypass graft
CK-MB: Creatine kinase-muscle/brain
CPB: Cardiopulmonary bypass
CTO: Chronic total occlusion
LAD: Left anterior descending artery
LCX: Left circumflex artery
RCA: Right coronary artery
SSS: Sick sinus syndrome
TFT: Thyroid function test
TSH: Thyroid stimulating hormone
TTE: Transthoracic echocardiography

## References

[1] Burch HB, Wartofsky L (1993). Life-threatening thyrotoxicosis. Thyroid storm. Endocrinol Metab Clin N Am.

[2] Galindo RJ, Hurtado CR, Pasquel FJ, Garcia Tome R, Peng L, Umpierrez GE (2019). National trends in incidence, mortality, and clinical outcomes of patients hospitalized for thyrotoxicosis with and without thyroid storm in the United States, 2004-2013. Thyroid..

[3] Akamizu T (2018). Thyroid storm: a Japanese perspective. Thyroid..

[4] Bish LT, Bavaria JE, Augoustides J (2010). Thyroid storm after coronary artery bypass grafting. J Thorac Cardiovasc Surg.

[5] Klein I, Ojamaa K (2001). Thyroid hormone and the cardiovascular system. N Engl J Med.

[6] Siu CW, Yeung CY, Lau CP, Kung AW, Tse HF (2007). Incidence, clinical characteristics and outcome of congestive heart failure as the initial presentation in patients with primary hyperthyroidism. Heart..

[7] Lee SM, Jung TS, Hahm JR, Im SI, Kim SK, Lee KJ, Lee JM, Chung SI (2007). Thyrotoxicosis with coronary spasm that required coronary artery bypass surgery. Intern Med.

[8] Langley RW, Burch HB (2003). Perioperative management of the thyrotoxic patient. Endocrinol Metab Clin N Am.

[9] Satoh T, Isozaki O, Suzuki A, Wakino S, Iburi T, Tsuboi K, Kanamoto N, Otani H, Furukawa Y, Teramukai S, Akamizu T (2016). 2016 guidelines for the management of thyroid storm from the Japan thyroid association and Japan Endocrine Society (first edition). Endocr J.

